# Admission to acute care hospitals for adolescent substance abuse: a national descriptive analysis

**DOI:** 10.1186/1747-597X-1-17

**Published:** 2006-07-10

**Authors:** Deena J Chisolm, Kelly J Kelleher

**Affiliations:** 1 Department of Pediatrics, The Ohio State University College of Medicine, Columbus, OH 43205, USA; 2 Center for Innovation in Pediatric Practice, Columbus Children's Research Institute, Columbus, OH 43205, USA

## Abstract

**Background:**

Use of alcohol and illicit drugs by adolescents remains a problem in the U.S. Case identification and early treatment can occur within a broad variety of healthcare and non-healthcare settings, including acute care hospitals. The objective of this study is to describe the extent and nature of adolescent admissions to the acute inpatient setting for substance abuse (SA). We use the Agency for Healthcare Research and Quality (AHRQ) 2000 Healthcare Cost and Utilization Project Kids Inpatient Database (HCUP-KID) which includes over 2.5 million admissions for youth age 20 and under to 2,784 hospitals in 27 states in the year 2000. Specifically, this analysis estimates national number of admissions, mean total charges, and mean lengths of stay for adolescents between the ages of 12 and 17 admitted to an acute care hospital for the following diagnostic categories from the AHRQ's Clinical Classifications Software categories: "alcohol-related mental disorders" and "substance-related mental disorders". Frequency and percentage of total admissions were calculated for demographic variables of age, gender and income and for hospital characteristic variables of urban/rural designation and children's hospital designation.

**Results:**

SA admissions represented 1.25 percent of adolescent admissions to acute care hospitals. Nearly 90 percent of the admission occurred in non-Children's hospitals. Most were for drug dependence (38%) or non-dependent use of alcohol or drugs (35%). Costs were highest for drug dependence admissions. Nearly half of admissions had comorbid mental health diagnoses. Higher rates of admission were seen in boys, in older adolescents, and in "self-pay" patients. Alcohol and drug rehabilitation/detoxification, alone or in combination with psychological and psychiatric evaluation and therapy, was documented for 38 percent of admissions. Over 50 percent of cases had no documentation of treatment specific to substance use behavior.

**Conclusion:**

General acute care hospitals have a significant and important opportunity to recognize, treat, and refer adolescents with substance abuse problems. These results suggest that inpatient facilities should develop and implement policies and processes to ensure that adolescent substance abusers admitted to their institutions receive appropriate care during the admission and appropriate referral to community care resources.

## Background

Use of alcohol and illicit drugs by adolescents remains a problem in the U.S. Although use rates have leveled and even decreased slightly for some substances over the past ten years, use rates among even the youngest adolescents are still concerning. Three of four teens have used alcohol (more than a few sips) by the time they finish high school and half have used alcohol prior to the 8^th ^grade. In addition, half of U.S. teens have tried an illicit drug by the time they finish high school with nearly 30 percent using a drug other than marijuana [1]. This substance use by adolescents is associated with negative outcomes including motor vehicle accidents, academic problems, family dysfunction, and criminal behavior [2,3]. In addition, early initiation of alcohol [4,5] and drug use [6] increases the probability of lifelong dependence.

While treatment for substance abuse disorders is generally provided within a well-defined segment of the health care community [7], case identification and early treatment can occur within a broad variety of healthcare and non-healthcare settings including primary care offices [8,9], emergency departments [10-14], educational settings [15], and the criminal justice system [16]. Research across these settings suggests that each can serve an important role in screening, treating, and referring adolescents with substance abuse problems but that time pressures and competing priorities sometimes limit their ability to serve this purpose.

To date, no research has been conducted on the role of acute care hospital admissions in the treatment of adolescents with substance abuse disorders. The inpatient setting is of particular importance because patients admitted to the hospital with substance abuse diagnoses are likely to have more severe or more complicated substance problems and because inpatients are in the hands of the health care setting for a longer period of time allowing for a variety of interventions and for appropriate referral planning [17].

The purpose of this paper is to describe the demographics, utilization, and cost of acute care inpatient admissions for adolescents with diagnoses of drug or alcohol related issues. Results of this analysis should prove useful in describing the impact of substance abuse on the inpatient care setting and should also help to quantify the opportunity for case identification, treatment, and referral within acute care hospitals. The results from this study should help to inform policy-making regarding opportunities for treatment initiation in the hospital setting and needs for improved the interfaces between acute care, behavioral health, and drug treatment settings.

## Materials and methods

### Data sources

This study was conducted using data from the Healthcare Cost and Utilization Project Kids Inpatient Database (HCUP-Kid) for the year 2000. The database provides detailed information on pediatric discharges (age 20 or less) from short-term general and specialty hospitals including children's hospitals. Discharges from hospital units of institutions, federal hospitals, psychiatric hospitals, and alcohol/chemical dependency facilities are excluded. The HCUP-KID includes over 2.5 million discharges from 2,784 hospitals in 27 states. Each record in the database represents a hospital discharge. Because not all states provide a unique patient identifier with HCUP-KID data, all analyses are conducted at the discharge, rather than the patient, level.

Weighting, based on hospital-level post-stratification on characteristics of urban/rural status, ownership/control, bed size, teaching status, U.S. region, and pediatric hospital status, is provided with the data to allow calculation of national estimates of utilization and cost. Detailed information on the content and design of the HCUP-Kid is published elsewhere [20].

### Case definition

Substance abuse-related acute care admissions were identified using AHRQ's Clinical Classification Software (CCS), a system which groups the over 12,000 ICD-9-CM diagnosis codes into 260 clinically meaningful categories [21]. Our case definition included admissions with a principal CCS diagnosis of alcohol-related mental disorders (CCS 66) and substance-related mental disorders (CCS 67). CCS 66 includes the ICD-9 diagnoses of alcoholic psychoses (291.xx) and alcohol dependence syndrome (303.xx) while CCS 67 includes the ICD-9 diagnoses of drug psychoses (292.xx), drug dependence (304.xx), and non-dependent abuse of alcohol or drugs (305.xx). For ease of reference, all cases meeting the above criteria are referred to as "Substance Abuse (SA) Admissions" for the remainder of this paper. Analysis was limited to adolescents ages 12 to 17.

It must be noted that the confidentiality and legality issues surrounding substance abuse diagnoses has led some states that participate in the HCUP program to restrict information provided on such admissions. The state of Pennsylvania requires that age be reported as the mid-point of a five-year age range for the diagnoses included in this study, in order to enhance confidentiality. This means that all children between the ages of 10 and 14 are coded as age 12. Given that this study includes adolescents between the ages of 12 and 17, Pennsylvania SA admissions for children aged 10 and 11 are included in our study. This will result in a slight overestimation of the national SA admission rates for adolescents between the ages of 12 and 17 but given the relatively low substance use in 10–11 year olds the impact should be small. The state of Texas requires that the age field for the diagnoses in this study be set to "missing," therefore, no SA admissions from Texas are included in our results. Because Texas also does not submit charge data to HCUP, AHRQ has developed survey weights for calculating national estimates excluding Texas, which are provided within the HCUP data. These weights will be used for this analysis; therefore the lack of Texas adolescent SA admissions should not bias national estimates.

### Analysis

The analysis describes hospital admissions for substance abuse across a number of demographic and utilization variables. Patient variables of age, gender, median income for zip code of residence, principal and secondary diagnoses, expected primary payer, total charges, and length of stay are taken directly from the HCUP-KID database along with hospital characteristic variables of location (urban/rural), and NACHRI hospital type (children's/general hospital/children's unit in a general hospital). This analysis also examines the inpatient utilization of Alcohol and Drug Rehabilitation/Detoxification (CCS Procedure 219) and Psychological and Psychiatric Evaluation and Therapy (CCS Procedure 218) for patients admitted for substance abuse and the discharge disposition of these patients.

We examined the distribution of substance abuse discharges within the demographic categories listed above. All frequencies are presented as national estimates calculated using the appropriate sampling weights. Category-specific admissions were described using two rates: admission rate per 1000 adolescent hospitalizations and, where possible, admission rate per 100,000 population based on the 2000 US Census [22]. Logistic regression was used to generate adjusted odds ratios (AOR) for hospitalizations with an SA diagnosis among hospitalized adolescents controlling for all studied variables. Significance of coefficients from logistic regression model was tested using a Wald Chi-squared test with 1 degree of freedom. We also examined the average cost and length of stay by diagnosis group. Means are presented as national averages using appropriate weights. All analyses used recommended procedures for calculating variances in the National Inpatient Sample [23]. Analyses were conducted using the SURVEYFREQ, SURVEYMEANS, and SURVEYLOGISTIC procedures in SAS ^© ^statistical analysis software [24].

The sample size for this study is extremely large; therefore, small, clinically-insignificant differences between groups are likely to be statistically significant using p-values of 0.05. For that reason and because of the number of comparisons being tested, we consider only p-values less than or equal to 0.01 to be meaningful.

## Results

### Patient characteristics

In 2000, an estimated 9,371 discharges with a principal diagnosis of substance abuse occurred nationally for youth aged 12 to 17. Given that there were an estimated 778,813 admissions in this period for youth between 12 and 17, this represents 12 of every 1000 inpatient visits in the age group. More than one third of SA admissions (38%) were coded as drug dependence and another third (35%) were for non-dependent use of alcohol or drugs. Over thirteen percent were coded as alcohol dependence syndrome and 11.6% were coded as drug-related psychoses. Alcohol-related psychoses were least common (1.7%) in this age group. Table 1 describes the demographic characteristics of admissions and compares rates across groups.

**Table 1:** Characteristics of adolescent substance abuse acute care admissions and admission rates by category

Characteristic^1^	Raw frequency	Weighted Total SA Admissions	Wgt % of Total SA Admissions*	SA Admission rate † (per 1000 adolescent admissions)	Pop. SA Admission Rate ‡ (per 100,000 population)
Total	3743	9371		12.0	38.8
Gender					
Male	2330	5927	63.4	19.7	47.7
Female	1397	3415	36.6	7.1	29.1
Age Group					
12–15	1164	3069	32.7	7.9	19.0
16–17	2579	6302	67.3	16.1	78.6
Primary Payor					
Private Insurance	1916	5067	54.8	12.0	
Government	989	2304	24.9	8.1	
Self-Pay	329	777	8.4	20.6	
Other	427	1105	11.9	38.4	
Zip Code Median Income ^a^					
1–24,999	346	729	7.9	9.1	
25,000–34,999	1071	2686	29.2	11.1	
35,000–44,999	1128	3330	36.2	15.5	
45,000 +	1111	2444	26.6	10.8	
Hospital Location					
Rural	540	1290	13.8	12.2	
Urban	3200	8073	86.2	12.1	
Hospital Type ^c^					
General Acute	3129	8002	86.7	14.8	
Children's Hosp.	57	159	1.7	1.9	
Children's Unit	485	1073	11.6	8.6	
Diagnosis Group ^a^					
291 – Alcohol Psychoses	66	155	1.7		
292 – Drug Psychoses	536	1,083	11.6		
303 – Alcohol Dependence	444	1,263	13.5		
304 – Drug Dependence	1,406	3,554	37.9		
305 – Drug/Alcohol Abuse	1,291	3,315	35.4		

^1 ^Individual frequencies may not add to total because of missing data (<5%) in any category

* Number of SA admission in group divided by total number of SA admissions

† Number of SA admission in category divided by number of any cause admission in category

‡ Number of SA admissions divided by number of individuals in population based on the 2000 US census

^a^Three-digit ICD-9-CM code

Table 2 presents odd ratios from a logistic regression model including all of the study variables. Admissions for boys were more likely to involve SA than admissions for girls, with 1.97 percent of male admissions having an SA principal diagnosis compared to 0.71 percent of female admissions (AOR = 3.3, 95% Confidence Interval (CI) 3.0–3.7). Admissions for older children were, also, more commonly SA (AOR = 2.1, 95% CI 2.0–2.4) than those for younger children.

**Table 2:** Logistic regression model for adolescent substance abuse admission

Characteristic	Coeff (SE)	Wald X^2^	p	Adjusted OR (95% CI)
**Gender**				
Female	Ref			
Male	1.20 (0.05)	618.43	<0.001	3.33 (3.02–3.66)
**Age Group**				
12–15	Ref			
16–17	0.76 (0.05)	248.65	<0.001	2.15 (1.96–2.37)
**Primary Payor ^a^**				
Private Insurance	Ref			
Government	-0.22 (0.05)	16.32	<0.001	0.80 (0.72–0.89)
Self-Pay	0.34 (0.08)	17.15	<0.001	1.40 (1.19–1.65)
Other	1.17 (0.07)	307.23	<0.001	3.23 (2.83–3.68)
**Zip Code Median Income ^b^**				
45,000 +	Ref			
35,000–44,999	0.36 (0.06)	40.13	<0.001	1.43 (1.28–1.59)
25,000–34,999	0.04 (0.06)	0.34	0.56	1.04 (0.92–1.16)
1–24,999	-0.16 (0.08)	4.24	0.04	0.86 (0.74–0.99)
**Hospital Location**				
Rural	Ref			
Urban	0.13 (0.06)	5.09	0.02	1.14 (1.02–1.28)
**Hospital Type ^c^**				
Children's Hospital	Ref			
General Acute	2.29 (0.14)	258.05	<0.001	9.94 (7.51–13.15)
Children's Unit	1.63 (0.15)	117.64	<0.001	5.09 (3.79–6.83)

†Odds ratios are adjusted for all variables listed in this table and are generated using weighted logistic regression including only admissions with no missing data on any independent variable (SA admissions = 8,342)

Self- pay (AOR = 1.4, 95% CI 1.2–1.6) and "other" payer admissions (AOR = 3.2, 2.8–3.7) were significantly more likely than private pay admissions to be for substance abuse while government pay admissions were slightly less likely. Admissions in general hospitals (AOR = 9.9, 95% CI 7.5–13.1) were considerably more likely to be for SA than those in Children's hospitals.

Half (50.9 percent) of principal SA admissions included a non-drug, mental health diagnosis coded as a secondary or lower diagnosis. Common mental health co-morbidities included conduct disorder and adjustment reaction disorder (39%), affective disorders (15%), and anxiety (10%).

### Comparative volume, charges and length of stay

Table 3 presents length of stay and charges by diagnosis category. Total charges for visits with principal diagnoses of substance abuse averaged $7,088 and length of stay averaged 8.9 days. Based on this sample, total inpatient charges for adolescent substance abuse admissions in the U.S. in 2000 exceeded $66 million. Visits for drug dependence were most expensive with average charges of $10,287 and average LOS of 14.5 days. The least expensive admissions were for alcohol psychoses with charges of $3,413 and LOS of 3.35.

**Table 3:** Mean charges and lengths of stay (LOS) by diagnostic group for admission with a principal diagnosis of substance abuse

Diagnostic Group*	Admissions	Mean LOS (Days)	Mean Total Charge
291 – Alcohol Psychoses	155	3.32 (1.97–4.66)	$ 3,413 (2,653–4,172)
292 – Drug Psychoses	1,083	4.11 (3.67–4.55)	$ 5,637 (4,897–6,376)
303 – Alcohol Dependence	1,263	8.35 (6.12–10.57)	$ 6,081 (3,894–8,269)
304 – Drug Dependence	3,554	15.29 (8.67–21.91)	$10,287 (4,601–15,973)
305 – Drug/Alcohol Abuse^a^	3,315	3.99 (1.60–6.38)	$ 4,657 (3,784–5,529)

*Admissions do not total 9371 because of rounding error within diagnostic groups

**Figure 1:**
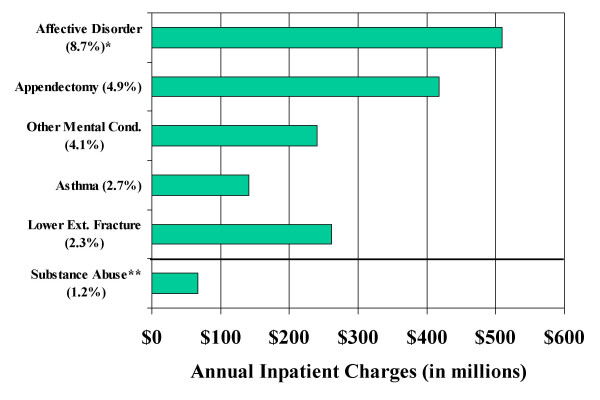
**Annual charges for adolescent substance abuse admissions compared to the top five CCS primary diagnosis categories by percent of admissions**. Estimated total U.S. hospital charges for adolescent admissions to acute care hospitals by primary CCS diagnosis category. * Percent of total adolescent admissions. ** Combines CCS 66 Alcohol-related mental disorders and CCS 67 Substance-related mental disorders

The combined principal CCS categories of drug-related and alcohol-related mental health conditions rank as the 26^th ^most common diagnosis category for adolescents. Excluding childbirth-related CCS categories these substance abuse diagnoses rank 17^th^. Figure 1 compares volume and charges for substance abuse to the top five principal CCS categories for adolescents: affective disorders (CCS 68), appendectomy (CCS 142), other mental conditions (non-psychotic) (CCS 74), asthma (CCS 128), and lower extremity fracture (CCS 230).

### Inpatient procedures and discharge disposition

Alcohol and drug rehabilitation/detoxification, alone or in combination with psychological and psychiatric evaluation and therapy, was documented for 38 percent of admissions. An additional six percent of admissions received psychological or psychiatric care alone. The remaining 56 percent of cases had no documentation of treatment specific to substance use behavior. Probability of drug/alcohol treatment varied greatly by principal diagnoses. While over 50 percent of admissions with diagnoses of alcohol psychoses, alcohol dependence syndrome, or drug dependence received alcohol/drug and/or mental health treatment; the treatment rate was less than 20 percent for admissions with diagnoses of non-dependent drug/alcohol use or drug psychoses. Table 3 summarizes treatments and discharge dispositions by principal diagnosis.

Most adolescent admissions for substance abuse resulted in routine discharges to home (82%), however, 7.6 percent were discharged to another type of facility, which could include skilled nursing facilities or inpatient drug treatment centers. Transfer to another type of facility was highest for patients with alcohol- or drug-related psychoses, 12.9% and 9.1% percent respectively. Overall, 6.8 percent of admissions left against medical advice (AMA). AMA discharges were highest for admissions with a principal diagnosis of drug dependence (11.4%). None of the admissions with a primary diagnosis of substance use died in hospital but this finding may simply be the result of the coding of proximal cause of death as the primary diagnosis in these cases.

## Discussion

There were over 9,000 admissions for substance abuse-related primary diagnoses at US acute care hospitals in the year 2000, with over 85% admitted to facilities not designated as Children's hospitals. Most admissions are related to drug dependency or drug/alcohol use. Not surprisingly, drug and alcohol psychoses, which are associated with long-term drug and alcohol use, were the least common. Comparison of substance abuse admissions to the top five non-childbirth-related principal admissions diagnoses by volume and charges shows that these substance abuse admissions do not trail far behind commonly researched adolescent conditions such as asthma.

Demographic characteristics of admissions were largely consistent with expectations. Our analysis found that admissions disproportionately included boys and tend to be clustered in older children. These finding are consistent with national epidemiologic data on drug abuse from the Youth Risk Behavior Survey conducted by the Centers for Disease Control and Prevention [25]. Also as expected, this study found a clear linkage between substance abuse and mental health diagnoses. Our estimate of 51% co-morbidity is consistent with that from other epidemiologic studies [9,26,27].

Substance abuse admission rates were significantly higher for self-pay patients and those covered by other insurers (i.e. CHAMPUS, Title V, Indian Health Service). The finding for other insurers is likely a reflection of the eligibility criteria for those insurance types while the self-pay finding may, in fact, be indicative of a lack of access to other types of treatment for the uninsured population.

Analysis of treatment patterns seems to suggest noteworthy opportunity for improvement in providing alcohol/drug and mental health care to adolescents admitted to short-stay general hospitals for substance abuse. Less than half of the admissions in this study had either alcohol/drug rehabilitation or psychological or psychiatric evaluation or treatment. Over 80% of patients with a principal diagnosis of non-dependent drug/alcohol use had no documented treatment. We note that it is possible that the seven percent of patients who were transferred to another type of facility upon discharge were transferred to facilities providing substance abuse treatment. It is also possible that patients are receiving brief interventions for substance abuse that are not coded in administrative data. Even given these possibilities, however, there appears to be opportunity for improvement in treatment.

The findings from this study point to specific actions for improvement of the care of adolescents with substance abuse disorders in the inpatient setting. The low rates of treatment recorded in administrative records among those admitted with a diagnosis of substance abuse raise serious concern. While it must be acknowledged that acute care hospitalization has increasingly emphasized medical stabilization and safety in an era of managed care, the very low rates of treatment initiation reported likely represent an opportunity for improvement in care. Other adolescent patients with chronic disorders subject to bouts of acute hospitalization, for example diabetes, receive intensive disease education and interventions during their inpatient admissions. Patients with substance abuse disorders deserve no less. Unfortunately, the failure to initiate treatment may be less due to a lack of initiative than to a lack of access to capable therapists with adolescent expertise. Severe shortages of specialty-certified and trained providers are reality in most of the U.S. and that is unlikely to be resolved soon.

Based upon these findings, we recommend that general and children's hospitals develop practice guidelines that clearly delineate a process for caring for adolescents presenting with substance abuse problems. These guidelines should outline the importance of brief intervention and of inpatient referral for substance abuse and mental health counseling. Recognizing that many hospitals may not have access to sufficient inpatient resources to address such referrals, guidelines should also provide contact information for resources available through other hospitals and in the community. Hospitals may, by improving their practices, play an important role in the continuing problem of adolescent substance abuse.

Because the HCUP-Kid database represents a "snap shot" of a single setting of care it is not possible to determine what if any healthcare these admitted patients had received prior to admission. We can however assume that these patients fall into two primary groups: 1) those who have not yet been clinically identified or referred to treatment, "the undiagnosed" and 2) those who have been clinically recognized with substance abuse problems but have not fully recovered, "the undertreated". For the "undiagnosed" population, the hospital admission serves as an opportunity for both problem recognition and treatment. Given that many adolescents, particularly the uninsured, rely on hospital emergency departments as their source for primary care, hospitalization may represent a teen's only interaction with the healthcare system [28, 29]. These admissions truly represent opportunities that should not be missed. On the other hand, in the "undertreated" population, the hospital serves not as an early intervener but as a "safety net". In these cases, the hospital should serve to reinforce messages from treatment programs and to refer individuals back into treatment, when necessary.

Several limitations of this study should be noted. First the ICD coding criteria for substance abuse were developed for adults. Because of this, some adolescents with clinically significant substance abuse problems will not meet the qualifications for diagnosis of substance abuse [26]. This would lead our study to underestimate the prevalence of substance abuse in admitted patients. Second, our study focuses on cases with a principal diagnosis of abuse. In truth, in some cases, the decision as to which diagnosis is coded as principal is a matter of judgment by the clinician and the coding staff, therefore focusing on primaries only may exclude some cases in which SA was as important as the listed principal diagnosis.

Another limitation to this study is the lack of a unique patient identifier on admissions. Because no unique patient code is consistently available, we cannot determine the number of individual patients represented in our 9,371 admissions. It is possible that these visits are generated by a relatively small number of patients being seen a large number of times. While this potential overestimate of the number of individuals admitted would not bias the estimated impact of substance abusers on the acute care hospital system, it would lead to an overestimation of the extent of undiagnosed or under-treated adolescent substance abusers.

A final limitation is the lack of appropriate data for race and ethnicity. According to AHRQ, 21% of states do not report race in their data submission and 22% of states that do submit race data have missing race data on over 20% of records. This reporting inconsistency between and within states make national racial estimates unreliable and therefore limits our ability to study potential racial differences which could be indicative of disparities in case recognition and treatment.

## Conclusion

More than one percent of acute care hospitalizations of adolescents have a substance abuse related principal diagnoses. While this may seem low it should be noted that the same data source estimates that appendicitis and asthma, conditions frequently discussed as important inpatient care diagnoses for children, represent only 4.7 and 2.3 percent of principal diagnoses, respectively. The inpatient admission for substance abuse, with its associated long length of stay, provides an excellent opportunity for healthcare providers to assess patients, offer brief interventions, provide counseling, and plan for follow-up care. In order to maximize the quality of care provided to adolescents with problem substance use, hospitals must develop treatment protocols and community linkages that facilitate long-term recovery.

## Competing interests

The author(s) declare that they have no competing interests.

## Authors' contributions

DJC was lead developer of the manuscript and was responsible for conception and design of the study, data analysis, and drafting of the manuscript. KJK participated in design and conception of the study and provided critical revisions for important clinical content. Both authors have given final approval of this manuscript.

**Table 4:** Treatment and Discharge Disposition by ICD-9-CM Principal Diagnosis

	Diagnosis*					
	Overall	291	292	303	304	305
Treatment						
Alcohol/Drug	37.1	55.2	16.4	58.2	56.2	14.4
Mental Health	6.0	0.9	2.2	2.5	11.8	2.5
Both	1.0	0.9	0.2	1.4	1.7	0.3
Neither	56.0	43.0	81.2	37.9	30.4	82.7
Discharge Disposition						
Routine	81.6	78.0	81.9	82.7	75.2	88.1
Transfer – Hosp	2.1	0	1.8	2.7	2.2	2.1
Transfer – Other Fac.	7.6	12.9	9.1	6.5	7.1	7.6
Home Health	1.9	0	1.0	1.1	4.0	0.3
AMA* *	6.8	9.1	6.2	7.0	11.4	1.9

*291 = Alcohol-related psychoses, 292 = Drug-related psychoses, 303 = Alcohol dependence syndrome, 304 = Drug Dependence, 305 = Non-dependent drug or alcohol use **AMA = Against medical advice;
